# Engaging community members to ensure culturally specific language is used in research: should I use gay, queer, MSM, or this other new acronym?

**DOI:** 10.1186/s40900-023-00463-0

**Published:** 2023-09-04

**Authors:** Kyle Rubini, Taim Al-Bakri, William Bridel, Andrew Clapperton, Mark Greaves, Nolan E. Hill, Max Labrecque, Richard MacDonagh, Glenndl Miguel, Shane Orvis, Will Osbourne-Sorrell, Taylor Randall, Marco Reid, Andrew Rosser, Justin Presseau, Elisabeth Vesnaver

**Affiliations:** 1 Local Advisory Group, Toronto Metropolitan University, Toronto, Canada; 2 Local Advisory Group, London, Canada; 3 Local Advisory Group, University of Calgary, Calgary, Canada; 4 Faculty of Kinesiology, University of Calgary, Calgary, Canada; 5 Local Advisory Group, Calgary, Canada; 6 School of Psychology, Ottawa Hospital Research Institute, University of Ottawa, Ottawa, Canada; 7 School of Epidemiology and Public Health, Ottawa Hospital Research Institute, University of Ottawa, Ottawa, Canada; 8 School of Epidemiology and Public Health, Ottawa Hospital Research Institute, University of Ottawa, Ottawa, Canada

**Keywords:** MSM, LGBTQ+, Community-based, Public health, Queer, Plasma donation, Sexual identity, Research practices, Ethics

## Abstract

Researchers often use terminology to define their participant groups that is rooted in a clinical understanding of the group’s shared identity(ies). Such naming often ignores the ways that the individuals who comprise these populations identify themselves. One oft-cited benefit of patient-oriented or community-engaged research is that language is local and relevant to impacted communities. This paper aims to contribute to the literature on how this local and relevant language can best be established. We ask how researchers can identify and implement accurate terminology, even when divergent perspectives exist within the communities involved. We draw from our experience with the Expanding Plasma Donation in Canada study, a community-engaged research study, which explored the views of people impacted by the “men who have sex with men” (MSM) blood donation policies in Canada. We describe the collaborative process through which we came to a consensual naming of this population, the challenges we faced, and a set of guiding principles we used to address them. We did not find an all-encompassing term or acronym that worked for all stages of research. Instead, we offer a set of guiding principles that can aid researchers engaging in a similar process: harm reduction, consent and transparency, collaboration and community involvement, recognition of missing voices, and resisting and/or restructuring oppressive standards.

**Supplementary Information:**

The online version contains supplementary material available at 10.1186/s40900-023-00463-0.

## Abstract (plain language summary)

The words and labels that researchers use to describe the communities they study does not always resonate with the actual members of those communities. Doing research in partnership with members of socially disadvantaged groups can help to ensure that the language used in the research is relevant, accurate, and respectful. Researchers studying issues related to men who have sex with men often struggle with knowing what term to use to describe this group of people. While many people may identify as “gay”, “queer”, “bisexual”, or any other term, there are many men who do not identify with these labels but also have sex with men. Previous research on this topic is usually focused on arguing that a specific term or acronym should be adopted. As part of a larger research program to support more inclusive plasma donation, the current paper describes the process our research team undertook to ethically describe this complex community of diverse men who have sex with men. Rather than choosing one specific label to describe the community members in all situations, we describe a set of guiding principles that can be used to help researchers flexibly navigate language depending on the situation and context.

**Supplementary Information:**

The online version contains supplementary material available at 10.1186/s40900-023-00463-0.

## Background

There is an enduring lack of consensus regarding the language and terminology used by health researchers and practitioners to describe and identify men who have sex with men (MSM) [1–8]. This paper aims to address the problem of finding an ideal standard-use term to describe all MSM within research projects. We contribute to this debate by claiming that no such ideal term exists and instead identify how ethical principles and contextual specificity can aid researchers when deciding which terms to use. These guidelines are based on our experiences conducting community-engaged research to support the development of interventions for blood plasma donation among communities previously excluded from donation based on their sexual behaviour, specifically MSM [9, 10]. We hope that our experiences can assist other researchers and practitioners to address and refer to community members in a way that identifies them accurately and correctly, as needed, in an ethical manner.

We begin with an overview of the history of the term *men who have sex with men* (MSM) and the debate regarding its use. We then provide background on the Expanding Plasma Donation in Canada Study and describe the collaborative process that we engaged in as a research team to identify suitable terminology for use in our work. We conclude that there is no all-encompassing term or acronym that will work for all stages of research. Instead, based on our experiences we propose a set of principles to consider when determining labels or terms to identify participant populations.

## Men who have sex with men (MSM)

The phrase “men who have sex with men” entered the HIV vernacular around 1990 and the acronym “MSM”, first used in 1994, has since been used as the standard language used within research communities [8, 11]. While this standardization may have been useful for research, the resulting widespread use of MSM has caused various levels of harm to community members, particularly affecting the most marginalized community members [2, 5, 11]. For example, MSM’s origins in HIV research has resulted in a gender imbalance regarding knowledge of sexual diversity issues [8]. As a result, MSM research (aside from HIV, such as mental health research) is disproportionately funded compared to research on women who have sex with women (WSW) and other sexual and gender minorities (SGM) [11].

Further, the term MSM term has harmed the transgender (trans) communities through ongoing and inconsistent misuse of the term to describe trans people [2, 5]. This has taken the form of conflating trans-feminine and/or nonbinary people with MSM, as well as excluding trans-masculine individuals who have sex with men [2, 5]. Further, research with Black MSM has found that treating MSM as one group neglects important aspects of identity that limits public health projects from reaching specific and most vulnerable communities [7].

Most critiques of using the term(s) MSM (and WSW) can be traced back to Young and Meyer’s [8] foundational paper that argued MSM (and WSW) should not be used as standard language but only in specific situations and contexts. Young and Meyer claimed that the behavioural focus of MSM was occasionally useful in specific contexts, but more often harmful based on their core premises that: (1) MSM dismisses self-determined identity labels, particularly among Black, Indigenous, and other people of colour (BIPOC), (2) the social aspects of sexuality are integral to understanding community sexual health, and (3) MSM obscures the most important elements of sexual behavior needed to understand how public health research and intervention can be enacted [8]. Young and Meyer assert that MSM and WSW are still useful and should not be discarded completely, but that the terms have become institutionalized and standardized in a harmful way that evades critical thought [8].

Young and Meyer further note that WSW and MSM have become racialized, such that the terms “gay” and “lesbian” are coded as white, and MSM/WSW are coded as various types of people outside the “mainstream/white” LGBTQ+ (lesbian, gay, bisexual, trans, queer, and other gender/sexual minority people) community, including BIPOC, poor people, sex workers, and people who inject drugs (PWID) [8]. Overall, Young and Meyer argue that labeling people as MSM and WSW despite contradictory self-labels is unethical [8]. They argue that by doing so, researchers are denying community members their ability to self-label, in turn they are denying their self-determination, disempowering these people, and making a harmful political statement about the limits of self-labeling as an LGBTQ+ person [8].

Many have argued that Young and Meyer’s recommendations are impractical and have proven to be unsuccessful based on the enduring popularity of MSM as a term [4, 6]. Much of the research contributing to this issue focuses on developing a new term to replace MSM, which has resulted in various terms being used based on researcher preference as well as inadvertently causing inconsistencies in database categorization [1, 3, 6]. Some of these terms and the arguments made for and against them are summarized in Table 1.

**Table 1:** Common terms used to identify men who have sex with men

Acronym	Meaning	Benefits	Limitations
MSM	Men who have sex with men	Does not use identity labels that do not match self-identities [6] Can describe a broad population [1] Historically benefitted men of colour who were less likely to self-identify as gay, due to historical social exclusion within gay communities, and sometimes preferring to identify as “on the down low” [3]	Inconsistent definitions of “men” and “sex” [6] Ignores all intersecting social identities and forces [6] Oversexualizes the population [6] Makes assumptions about what constitutes “sex” [6] Not a label that many people self-identify with [1] Historic misuse with transgender populations [1, 2, 5] Disempowers community members by ignoring their self-labels [8]
GBQ	Gay, bisexual, and queer men	Empowers community members by honouring self-identities [1]	Excludes straight-identifying and other men who do not identify as gay, bisexual, or queer but still have sex with men
-	Self- identified labels	Empowers community members by honouring self-identities [1]	Sometimes impractical during the research process [4, 6]
SMM	Sexual minority men	More useful as a search term than MSM [6] Describes a broader population than MSM [6]	Not a label that many people self-identify with [1] As with MSM, SMM disempowers community members by ignoring their self-labels [1] Not necessarily more inclusive for trans men than MSM [1] Using the term “minority” may perpetuate imbalanced power dynamics [1] The term “sexual minority” is broad/vague and could be misinterpreted to include any non-heteronormative communities such as fetish communities and others [3] Centralizes whiteness [3]

## MSM, blood and plasma donation, and the Expanding Plasma Donation in Canada study

In response to the AIDS crisis, many countries implemented criteria to restrict blood and plasma donation by groups identified at the time to be at high-risk of contracting HIV [9]. MSM were one of these groups. In Canada, MSM were banned from donating if they had had sex with a man even once since 1977 (the date believed to be the first appearance of AIDS in North America). In 2021, Canadian Blood Services implemented a small pilot program whereby some “MSM” who were at low to no risk of HIV infection could donate plasma. The *Expanding Plasma Donation in Canada Study* was designed to support the implementation of the pilot program and involved understanding the barriers to implementing new criteria among staff and to donating among impacted communities and developing interventions to address these barriers [10]. The study was rooted in community-engaged research methods and involved community members who identify as being impacted by MSM donor criteria[12]. As such, all community members involved identified as a type of man who has sex with men. Two local advisory groups (LAGs) of 6–8 members were formed in each respective Canadian city where the pilot project was being run: London, Ontario and Calgary, Alberta. Committee members were recruited through online advertisements and word-of-mouth snowball techniques. They shared the experience of being excluded from blood and plasma donation, and the desire to help progress these policies. Most members also shared the motivation to donate blood.

The London and Calgary LAGs met online for monthly meetings from Dec 2019 to October 2021 and Dec 2020 to October 2021 respectively. The groups then joined and met in combined meetings from November 2021 to July 2022. LAG members contributed to a Terms of Reference agreement outlining their involvement which included annual honoraria. During meetings, LAG members met with research institute-based team members to discuss stages of the research process and provide feedback based on their lived experience to ensure the project was conducted in a way that was as culturally competent as possible. Decision making was shared as much as possible to ensure that findings and outputs would benefit impacted communities. LAG members contributed to research question refinement, development of data collection tools, development of recruitment strategy and materials, recruitment, interpretation of findings, intervention development and dissemination (see https://expandingplasma.ca for one intervention co-developed with LAG members).

LAG meetings were facilitated by a member of the research team (EV), and open discussion was encouraged. These meetings maintained an organic balance of relationship building and project focus. We often began with a group icebreaker question and general personal updates. LAG members often self-regulated these social interactions such that topics were generally non-controversial and/or not emotionally triggering.

Regarding research-related discussions, LAG participants often discussed their perspectives openly. They relied on their lived experiences, conversations they had had with community members outside the LAG, and for some, their professional experiences. Certain themes frequently arose, particularly the issue of what term would be used to describe the community of MSM or the communities impacted by the MSM donor criteria as there was disagreement among members. With no clear best term available in the literature and consistent with the importance of open dialogue, mutual learning, and participatory decision making in community-engaged practices [12], the postdoctoral fellow who led the LAG meetings suggested a dedicated discussion on the topic where they would collectively decide the language that the research team would move forward with.

This purpose of this paper is to describe the collaborative process through which we came to a consensual naming of this population, the challenges we faced, and a set of guiding principles that encompass how we addressed them. The paper was led by KR, a member of the LAG, who is also a PhD student in Communication and Culture at Toronto Metropolitan University, and was supported by EV. All other members of the LAG (TA-B, WB, AC, MG, NEH, ML, RM, GM, SO, WO-S, TR, MR, AR) contributed to the collective decisions on language. JP is the principal investigator of the *Expanding Plasma Donation in Canada Study*.

## Main text

### Collaborative process

The research institute-based team had initially used the term MSM guided by the scholarly norm in blood donation research literature, but shifted to GBMSM (gay, bisexual, and other men who have sex with men) after a LAG member (WO-S) circulated the Young and Meyer paper [8]. GBMSM acknowledged both self-determined identities and behaviour and was used during our data collection in London, Ontario. However, our Calgary advisors felt that “Men who have Sex with Men” was more succinct and inclusive particularly since GBMSM “others” men who have sex with men who don’t identify as gay or bisexual. As a result, we used recruitment materials in Calgary that prioritized MSM or only using labels such as gay, bisexual, pansexual, and queer. When findings from the project were nearing the point of dissemination, both groups met to come to a decision on consistent terminology to disseminate the research findings.

A dedicated meeting was held on November 23, 2021, where members of both the Calgary and London LAGs were invited to join and discuss the topic of MSM and language at a deeper level and to decide collectively how the project team would refer to MSM in project outputs. The group split into two breakout rooms each with Calgary and London LAG members and a member of the research team to take notes. The group came together after to share conclusions from each group and have a larger discussion. A member of the research team (EV) analyzed the notes taken and presented some suggestions in a collaborative google document that was circulated and iterated among LAG members. The following summarizes these discussions while also including points raised during other meetings or in subsequent emails.

Since this consensus process arose from a pre-existing research group, the LAG members in question were not chosen to reflect the diverse range of community member identities that would be ideal for this process. All LAG members identify as cisgender men and either gay, bisexual, queer, or pansexual. Twelve (of 14) identify as white. We did not collect socio-demographic details about LAG members beyond gender, sexual orientation, and ethnic or racial identity. The concluding guiding principles from this process are limited based on LAG members’ identities, lived experience and perspectives. We encourage other researchers to build on this work and further contribute to the framework developed within this paper.

### Lively debate with no consensus

While some LAG members rejected the term MSM based on its clinical connotation and distance from self-labels, others embraced the term for its broad reach. Those in support of using MSM noted that the term was “simple” and could be “explained easily”. The term’s international ubiquity was also beneficial when considering reaching particular audiences and wanting our research (and consequently the voices of our participants) to be disseminated widely. Interestingly, other acronyms such as SMM were not treated much differently than MSM as the acronyms were often met with similar arguments.

The word “queer” was more favourably embraced by younger members of the LAG while older members often felt that the term did not fit most of the community and that many individuals continue to be harmed by the word as a result of its historical derogatory uses. Many LAG members noted that they were aware of the word “queer” being reclaimed but argued that that did not erase the damage it had caused, change the emotional reaction many still have to the word, or alter how many people within the community identified themselves.

The acronym GBQ received a more positive response from most LAG members as it included “queer” but also the more traditional self-labels of “gay” and “bisexual”. Many noted that it would be easy to combine GBQ and MSM to make an acronym that was inclusive but also allowed for more specific self-labels. Also, this combined acronym GBQMSM (gay, bisexual, queer, and all men who have sex with men) could use the word “all” rather than “other” to strategically include all men who do not identify within the community. This acronym also builds off GBMSM (gay, bisexual, and other men who have sex with men), which is becoming more commonly used within HIV education work.

In reference to GBMSM, one member stated that he often uses this acronym within his personal advocacy work but continues to be flexible and adjusts his language based on the audience/group he is speaking to. However, it was noted that mainstream media outlets are resistant to using the term “men who have sex with men”. Another acronym, 2SGBTQM+ (two-spirit people, gay men, bisexual men, trans masculine people, queer men, men who have sex with men, plus other people outside the gender binary who have sex with men) was recommended as it was most inclusive and specific, while also resembling popularized acronyms such as LGBTQ+.

One member argued that no acronym will ever be truly inclusive and that a complex acronym will reduce the accessibility of our work, particularly in the context of the media. Another member countered that they did not want to cater to a “lazy majority” and felt it was our role to challenge any resistance to the use of self-determined terms. Given the challenges with identifying who exactly is impacted by blood donation restrictions, phrases such as “people or communities impacted by the policies” resonated with many in its simplicity and efficacy, while allowing for people to self-identify.

When we discussed the possibility of a more specific term, it was suggested that we find something that focused on sexual behaviour over gender as it would be most helpful in the context of blood donation policy where behaviour should be prioritized over sexual orientation/desire. An actual term for this was not decided upon, though the humourous stand-in of “anal pals” was used to guide discussion. LAG members noted that a new term would likely cause further confusion within the already complex discourse of sexual language, that it would likely not be easily searched in databases, and that the media would likely not respond well to a new term considering mainstream media’s demonstrated resistance to the word “queer”.

We found that personal preference was occasionally a factor in deciding a term, but that the situational context and purpose of the term was most important. Thus, we decided that certain terms would be used in certain situations, but particular protocols would be followed in order to ensure harm reduction. Such protocols included: a focus on transparency, meaning that the research team—including the LAG—would always know which term would be used when, and why as well as allowing for some flexibility in that different terms would be used at different stages of the research process.

The most prominent concern was the difficulty of identifying men who identified as straight, “without a label”, or otherwise, but still engaged in sex with other men. Broad terms such as “queer” or “rainbow community” do not include this group of men. No suitable alternative for MSM was found through discussion or research.

One member stated that “there isn’t enough language to include everyone”, since any term we discussed always excluded a group of people. While past research has encouraged the issue of language terminology to be resolved through communication with community members [8], we found that similar debates and lack of resolution continued to emerge. Many LAG members echoed Malebranche’s argument that language discourse can never fully be resolved and focusing too heavily on this issue can detract from more prominent issues that are impacting these communities in a greater way [3].

Even if there had been resolution, one LAG member astutely noted that “it's important to acknowledge the potential whiteness of a term decided upon by a small group that was limited by not having more diverse representation of visible, sexual, and romantic minorities.” Generally, most members agreed that simplicity and harm reduction should guide decisions about language use. One LAG member summed up the issue succinctly by stating that their most important question is often, “can I find myself in this information?” and that researchers should let this question guide their decisions about language.

This issue of finding yourself in a set of information is particularly relevant to BIPOC MSM, and whether they can find themselves in this language. This is further complicated when considering how the research community has affected terminology. For example, while MSM has historically been a way to unofficially codify Black MSM, this does not necessarily mean that Black MSM actually feel recognized by such a term [8]. Further research should examine racialized MSM and other sub-communities in order to better understand how we can help them find themselves in recruitment information and other research-related materials.

A particular challenge in the determination of one all-encompassing term was a qualitative difference in the “who” we were attempting to recruit for our research study and the “who” blood operators considered to be MSM. During the study period, the criteria related to sex between men applied to individuals who were assigned male at birth who were sexually active with individuals assigned male at birth unless the individuals in question had undergone lower genital gender affirming surgery. Thus, these criteria did not only impact men, but also non-binary and trans individuals. Since the purpose of the larger research study was to develop interventions to support plasma donation in the new 2021 pilot program, we had recruited participants who identified as men (cis and trans) as the policy and screening remains gendered along the binary [13] and gendered language would be used by Canadian Blood Services (CBS) for recruitment of new donors. Although not the targets for our interventions, many who do not identify as men are nonetheless impacted by these policies and we needed more inclusive language to refer to these communities when describing the policies and their impacts in our outputs.

As we worked through our discussions and realized that we couldn’t identify one term to accurately identify our participants and the broader communities impacted by the policies, we shifted our practice again. We accepted that we would need more than one term, to both reflect our participants and the broader communities impacted by the MSM donor restrictions. For our participants, we used ‘gay, bisexual and other men who have sex with men (gbMSM)’ to reflect our language of recruitment and the self-selection and consent of those terms that participants had already engaged in to participate in our study [14].

For the broader communities impacted by the MSM donor restrictions, our write-ups currently utilize a combination of “MSM”, “2SGBTQ” (two-spirit people, gay men, bisexual men, trans people, and queer men), and “all impacted communities”[10]. This was more inclusive of the broader communities impacted by the policy but also included the original gbMSM language that our participants had already consented to (since they are also members of the broader communities). Using both MSM and an acronym of identities broadened the scope of who would be able to find themselves in this term. Keeping MSM benefitted not only inclusion of those who identify in this way, but remains recognizable for public health or scientific audiences. The acronym 2SGBTQ+ is also increasingly being used by men’s health clinics. Please see Additional file 1 for the language that we agreed upon for outputs through this process and our rationale for each decision. Although there was not complete consensus on the ‘best’ terms, there was consensus that the process was thoughtful and the result sensitive, if not perfect.

Given that community description language was not the main concern of the *Expanding Plasma Donation in Canada* research project, the group agreed to move forward with these terms, but continue working on problem-solving this issue through various avenues. As a result, LAG members led this paper to document and share our discussions while proposing an alternative process. Certain things can be done to mitigate the issues of whatever language is used. In our circumstance, we decided to (at the minimum) include footnotes in our write-ups to acknowledge the limitations of the language chosen and to specify the people that our terminology is meant to include. We conclude that there is no acronym or term that will fit all situations, but that this language should be chosen deliberately and ethically. This can be done by considering a set of guiding principles designed to ensure harm reduction and practical use within the field.

### Proposed practices and principles

Self-identifying within the LGBTQ+ community is a particularly sacred aspect of community practice [8]. Contemporary research needs to restructure its approach to labelling LGBTQ+ community members given the historic legacy of harmful language being ascribed to the community [8]. Such terms were created and reified by scientific nomenclature (tainted, abnormal, degenerate, inverted, sexual psychopaths) that vilifies, pathologizes, and denigrates community members to this day [8]. Sexual minority communities have had to fight to overcome these labels and worked to create new terms (two-spirit, transgender), and reclaim harmful words (queer) they can celebrate and use to self-identify [8].

Based on our experience working with community members and health researchers, our conclusions align with Young and Meyer’s original argument that there is no singular acronym or term that will always work [8]. Rather, a reflexive and critical approach must be used when working with these communities and choosing which language to use when describing them [4, 6, 8]. We have developed a set of practical guiding principles based on our group discussions, Young and Meyer’s four research labeling principles, and the Montreal Ethical Principles of Inclusive Research [8, 15].

These guiding principles were our solutions to the challenges we faced in coming to consensus on appropriate language to describe the study’s participants as well as the broader group of people who are impacted by the MSM donor criteria. These principles were developed primarily by the LAG members through the writing of this manuscript and retrospective reflection on our processes. We reviewed transcripts of our discussions and linked the major themes with the actual practices we used as a group throughout our work with the *Expanding Plasma Donation in Canada Study*.

We offer these guiding principles for labelling LGBTQ+ community members as a starting point for community researchers and practitioners to consider. These principles are limited by the identities and perspectives of our research team and the context of the project for which they were developed. Furthermore, language evolves over time as should these practices. As such, we welcome all contributions and updates to this guide that may help community members be labeled and identified in the most humane and ethical way possible.

The first principle, “harm reduction”, encourages researchers and practitioners to consider all potential harms that could result from the labels used. Harm reduction should be prioritized over the standardized use of any one label. Decisions about language should be made on a case-by-case basis to ensure that the label used causes the least amount of harm to those groups of people being described. For example, although using self- identified labels is generally best-practice, one harm that may ensue is reduced reach of the research and consequently of the voices of participants. Adopting some flexibility in labeling such that participant self-identifiers are primarily used, while also including other labels (e.g. MSM) for certain instances, such as key terms for searchable database accessibility, may be one strategy to reduce harm.

The second principle, “consent & transparency” ensures that participants are aware of the words and labels that will be used to describe them and the rationale for doing so. This is particularly important if flexibility for certain situations is needed to reduce harm (see above principle). This gives participants agency to maintain or withdraw their consent to participate with full knowledge of the labelling strategy.

The third principle, “collaboration & community feedback”, describes a genuine partnership between participants and researchers/practitioners. Such a partnership would require gathering information from a diverse range of community members regarding the actual language/labels that such community members are most and least comfortable with. Such data should also include the rationale behind the use or avoidance of terms to better understand the emotional stakes of each label.

The fourth principle is intrinsically tied to community feedback, “recognition of missing voices” is about identifying the representation of the input received and at a minimum acknowledging this. When possible, researchers should seek community feedback from those people that are not already doing so. Researchers should assess whether their participant groups accurately reflect the diverse range of people within their target population. This is a complicated but necessary task that may require specific outreach to sub-communities, reduction/elimination of barriers to engage, equity assessments, and other related tasks.

The final and most important principle is often most likely to extend beyond any particular research project. “Resisting and/or restructuring oppressive standards” encourages researchers to be creative and occasionally radical in their approach to research and community engagement. This principle reminds researchers/practitioners that research standards are constantly evolving, and any project’s approach to these complex issues of ethically labelling participant groups can be used to help other researchers as well as the community members that are being researched. Table 2 describes all five principles in a general sense to reflect their application throughout various stages of the research process.

**Table 2:** Guiding practices and principles for labeling LGBTQ+ participants

Guiding practice/principle	Details
Harm reduction	Use the acronym or term that causes the least amount of harm at each stage of the research/engagement process
Consent & transparency	Make it clear to all people involved what language you are using at each level and why it may be different in each situation. If the language you used will change or be coded differently at any level, make that clear to your participants and provide rationale for doing so
Collaboration & community feedback	Use a horizontal (if not bottom-up) approach of developing language with community members while recognizing the limitations imposed by current research practices
Recognition of missing voices	Develop strategies to account for those communities that are not part of the process for whatever reason, this may include MSM, trans people, BIPOC, disabled people [16], PWID, or others. This means that demographic data about participants should be monitored to account for those who may be excluded by existing research protocols. Aim to resolve these issues through direct outreach when possible
Resisting and/or restructuring oppressive standards	Recognize, challenge, and restructure existing oppressive research practices and standards to make way for new forms of language and community recognition. This may include challenging the use of oppressive standards and proposing alternatives. Such areas to examine may include but are not limited to: the peer review process for publications and grant adjudication, media/social media dissemination and engagement, project meeting procedures, and hiring processes

## Conclusions

The ongoing debate about MSM and other terms used will only continue to detract from more prominent research issues until researchers accept that no single acronym or term can be used for all occasions. Within this paper, we have reviewed the various terms and acronyms suggested for research use, and the arguments for and against using such terms. By drawing on our research team’s discussions and findings, we were able to discuss how process and flexibility could be used in order to better use language that is put forth by the community members themselves. As a logical extension of this work, we proposed a set of guiding principles that researchers and community practitioners can use in their work when deciding on language to use in specific situations. This paper also offers a detailed example of how a community-engaged research project fostered participatory decision making on a small but critical component of the research project.

### Supplementary Information

**Additional file 1**. Decisions and rationale of the Expanding Donation in Canada study team regarding language to describe the communities impacted by the MSM donor criteria.

## Data Availability

Not applicable.

## Abbreviations

2SGBTQ: Two-spirit people, gay men, bisexual men, trans masculine people, and queer men
2SGBTQM+: Two-spirit people, gay men, bisexual men, trans masculine people, queer men, men who have sex with men, plus other people outside the gender binary who have sex with men
AIDS: Acquired immunodeficiency syndrome
BIPOC: Black, Indigenous, and people of colour
LAG: Local advisory group
HIV: Human immunodeficiency virus
LGBTQ+: Lesbian, gay, bisexual, transgender, queer, and other sexual/gender minority people
MSM: Men who have sex with men
PWID: People who inject drugs
SMM: Sexual minority men
WSW: Women who have sex with women
