# Supplementary material for: Engaging community members to ensure culturally specific language is used in research: should I use gay, queer, MSM, or this other new acronym?
Source: Res Involv Engagem. 2023 Sep 4;9:75. doi: 10.1186/s40900-023-00463-0 (PMC10478417; doi:10.1186/s40900-023-00463-0)
Supplement: Supplementary file 1 — Additional file 1. Decisions and rationale of the Expanding Donation in Canada study team regarding language to describe the communities impacted by the MSM donor criteria. [file 40900_2023_463_MOESM1_ESM.docx]

**Expanding Plasma Donation Study in Canada**

Consensus document on language for use for study outputs

Contributors: Kyle Rubini, Taim Al-Bakri, William Bridel, Andrew Clapperton, Mark Greaves, Nolan E. Hill, Max Labrecque, Richard MacDonagh, Glenndl Miguel, Shane Orvis, Will Osbourne-Sorrell, Taylor Randall, Marco Reid, Andrew Rosser, Justin Presseau, & Elisabeth Vesnaver

Challenge:

Population impacted by Canada’s MSM donor policy is different from the population included in our study. Need to identify appropriate language for each population.

Collective goals:

-Use language that resonates with, and is understandable by our audiences

-Be precise and inclusive in our language

-Challenge the current simplistic language in use (CBS, Media, Scientific Articles)

Result

We were unable to come to consensus on terms. The group came to consensus that the ways of referring to the populations as indicated in the table was inclusive, thoughtful, and sensitive. There was a belief that consensus on optimal language was unlikely, but the process resulted in terms that the group found acceptable. In the below table, we outline the resultant decisions for the terms we will use in given situations or scenarios. Any further explanatory notes or justification are provided in the “Notes” column. In some cases, it was agreed that due to the imperfection of the language agreed upon, further context or rationale would need to be provided in our outputs. In order to standardize this language, we included these additional elements in the ‘language we will use” column.

| **Situation/Scenario** | **Language we will use** | **Notes** |
| --- | --- | --- |
| **To describe our recruitment** | We used social media and word of mouth to recruit cis and trans gay, bisexual, queer, pansexual men and men who have sex with men.  Survey participants answered a screening question in order to complete the survey. Participants were asked: “In this research study, we are looking to get the views of men* who have sex with men who are at least 18 years of age and live in  Calgary/London and surrounding area – Does this describe you? *Cis and trans men are eligible”.  Individuals who did not meet the screening criteria for inclusion in the survey but felt they were impacted by the proposed pilot program were invited to email the research team to discuss eligibility. No participants contacted us to be included. Several participants identified as men in the screening but later selected non-binary for their gender. This recruitment approach inappropriately required non-binary donors who felt impacted by the pilot program to self-select as men in the initial screening in order to have their voice heard through the survey. Trans feminine individuals were not targeted through recruitment although many would be impacted by the criterion. Future research should use more inclusive recruitment and screening methods. | This is the language we actually used to recruit and thus we must report this in manuscripts.  We can acknowledge the shortcomings of this approach, and put forward more inclusive approaches for future research. |
| **To describe our participants** | Interviews: Men who self-identified as gay, bisexual or as having sex with men^1^  Surveys: Individuals who self-identified as gay and bisexual men or as men who have sex with men  -Cis and trans men and non-binary individuals who felt impacted by the men who have sex with men (MSM) blood donation criterion.  ^1^ Cis and trans men were recruited to participate. Trans feminine individuals were not included in the study although many are impacted by the criterion (see note on policy)  Also describe other demographic information (age, racial identity etc). | We use “self-identified” because participants responded to our ads which used this language. We chose “self-identified” rather than “identified” in this case to highlight the act of self-selection for the study and distinguish it from the terms someone might use to describe their identities. In any paper, we will acknowledge that neither this sample, nor our recruitment strategy was inclusive of all people impacted by these policies.  In our interview sample, all identified as male/masculine when asked their gender.  Survey included men, transmen/masculine, non-binary men, non-binary individuals |
| **To describe the policy** | Blood Ban, Blood donation criterion for men who have sex with men (MSM).  In Canada at the time of writing, this criterion applied to individuals who were assigned male at birth who were sexually active with individuals assigned male at birth unless the individuals in question have undergone lower genital gender affirming surgery. This policy impacts many communities including individuals who identify as two-spirit, gay, bisexual and queer men, trans feminine and non binary persons, or as men who have sex with men. It is our view that the criterion is inappropriately named. It conflates gender and sex, erasing the experience of individuals who do not identify as men yet are impacted by the criterion. MSM is widely used in public health, often inaccurately. Among individuals identifying as men who are targeted with this term, MSM is indeed the right term for some; for others, the term obscures the complex interconnecting social, cultural and behavioural realities experienced by individuals)^2^. Among individuals who do not identify as men, yet are screened using this criterion, the term is harmful. We have aimed to be inclusive of the ways that our participants and our community research partners wish to be identified, acknowledging the limitations of our decisions by the range of racial representation, gender expression and romantic relationships represented in our research.  ^2^ Young RM, Meyer IH. The trouble with “MSM” and “WSW”: Erasure of the sexual-minority person in public health discourse. American Journal of Public Health. 2005;95:1144-9. | Preference to describe policy first before describing the population impacted whenever possible. This places responsibility for this categorization on the policy and the institutions that uphold it. It also helps to anchor our work at this time in history before the policy change. |
| **To describe the population group that has been impacted by this policy** | Communities impacted by the MSM criterion  “Impacted Communities”  Community impacted by the MSM criterion including but not limited to gay and bisexual men | Preference to describe policy first before describing the population impacted whenever possible. This places responsibility for this categorization on the policy and the institutions that uphold it. |
|  | In the case we need to describe this population before describing the policy:  Two-spirit, gay, bisexual and queer men, trans women and non binary persons and men who have sex with men (2SGBTQ+/MSM)  **OR**  Men who have sex with men including but not limited to gay, bisexual, and queer men and two-spirit, transgender, and non-binary individuals (MSM/2SGBTQ+) | 2SGBTQ+/MSM aligns with the All Blood is Equal Campaign. It includes both the MSM and an acronym of identities – both of which are recognizable. Keeping MSM is both inclusive for those who identify this way and very recognizable for public health or scientific audiences. 2SGBTQ+ is increasingly being used by men’s health clinics.  Because many argue that identity labels such as gay, bisexual, and queer are largely claimed by white individuals, whereas MSM tends to be claimed more often by racialized men in North America, we should consider how the order centres/preferences groups/experiences. One problem with this acronym is that the letters don’t go in the order of the text. However, the acronym is more recognizable which meets one of our goals. |
| **To describe the population that is eligible with MSM plasma program** | Newly eligible donors who were previously excluded due to the MSM criterion |  |
